# The Anti-Inflammatory and Antibacterial Basis of Human Omental Defense: Selective Expression of Cytokines and Antimicrobial Peptides

**DOI:** 10.1371/journal.pone.0020446

**Published:** 2011-05-24

**Authors:** Abhijit Chandra, Ritesh Kumar Srivastava, Mahendra Pratap Kashyap, Raj Kumar, Rajeshwar Nath Srivastava, Aditya Bhushan Pant

**Affiliations:** 1 Department of Surgical Gastroenterology, Erstwhile KG Medical College, CSM Medical University, Lucknow, India; 2 Indian Institute of Toxicology Research, Lucknow, India; 3 Council of Scientific & Industrial Research, New Delhi, India; 4 Department of Basic Sciences, The Commonwealth Medical College, Scranton, Pennsylvania, United States of America; 5 Department of Orthopaedic Surgery, Erstwhile KG Medical College, CSM Medical University, Lucknow, India; Charité, Campus Benjamin Franklin, Germany

## Abstract

**Background:**

The wound healing properties of the human omentum are well known and have extensively been exploited clinically. However, the underlying mechanisms of these effects are not well understood. We hypothesize that the omentum tissue promotes wound healing via modulation of anti-inflammatory pathways, and because the omentum is rich in adipocytes, the adipocytes may modulate the anti-inflammatory response. Factors released by human omentum may affect healing, inflammation and immune defense.

**Methodology:**

Six human omentum tissues (non obese, free from malignancy, and any other systemic disorder) were obtained during diagnostic laparoscopies having a negative outcome. Healthy oral mucosa (obtained from routine oral biopsies) was used as control. Cultured adipocytes derived from human omentum were exposed to lipopolysaccharide (LPS) (1–50 ng/mL) for 12–72 hours to identify the non-cytotoxic doses. Levels of expression (mRNA and protein) were carried out for genes associated with pro- and anti-inflammatory cytokine responses and antibacterial/antimicrobial activity using qRT-PCR, western blotting, and cell-based ELISA assays.

**Results:**

The study shows significant higher levels of expression (mRNA and protein) of several specific cytokines, and antibacterial peptides in the omentum tissues when compared to oral sub-mucosal tissues. In the validation studies, primary cultures of adipocytes, derived from human omentum were exposed to LPS (5 and 10 ng/mL) for 24 and 48 h. The altered expressions were more pronounced in cultured adipocytes cells when exposed to LPS as compared to the omentum tissue.

**Conclusions/Significance:**

Perhaps, this is the first report that provides evidence of expressional changes in pro- and anti-inflammatory cytokines and antibacterial peptides in the normal human omentum tissue as well as adipocytes cultured from this tissue. The study provides new insights on the molecular and cellular mechanisms of healing and defense by the omentum, and suggests the potential applicability of cultured adipocytes derived from the omentum for future therapeutic applications.

## Introduction

The human omentum has been classically regarded as the abdominal policeman [1] with instances of its reaching and plugging inflamed intra-abdominal organs [2]. Recent clinical evidence has suggested spontaneous sealing of intestinal perforation in premature neonates by the omentum alone, where just aspiration was adequate [2]. Omentum has been shown to secrete many biological agents including *vascular endothelial growth factor* (VEGF), different other growth factors, and cytokines [3], [4], [5]. The omentum milky spots are conglomerates of macrophages responsible for its local immune response and anti-inflammatory properties [4], [6]. In the omentum, the non adipose cells of the stromal vascular fraction, preadipocytes, and macrophages are thought to secrete cytokines [7], [8], [9]. However, recent data suggests the involvement of adipocytes in the inflammatory response [10], [11]. Even though the role of omentum tissue in wound healing and injury repair is well established, the cellular and molecular mechanisms underlying these properties remain unclear.

We hypothesize that the omentum tissue promotes wound healing through anti-inflammatory pathways, and because the omentum is rich in adipocytes, the adipocytes may modulate the anti-inflammatory responses [12], [13]. The omentum may also possess an intrinsic direct antibacterial activity of its own [5]. Thus, the present studies were carried out to determine the levels of expression of selected pro- and anti-inflammatory cytokines (mRNA and protein) and antibacterial peptides genes in human omentum tissue derived from normal, disease-free human subjects, and cultured adipocytes obtained from these tissues. Further, the expression and up-regulation of these genes were also assessed in primary adipocyte cultures derived from omentum exposed to bacterial endotoxin. Our results show that adipocytes modulate the expression of several pro- and anti-inflammatory cytokines and anti-microbial peptides.

## Results

### Effects of LPS (lipopolysaccharide from *Escherichia coli*) exposure on cell viability

To determine the non-cytotoxic doses of LPS exposure to cultured adipocyte cells derived from human omentum tissues, we carried out MTT assay. The results of cytotoxicity assays are shown in Figure S1. It is evident from Figure S1 that there is no significant reduction in cell viability due to treatment of LPS at lower concentrations (≤20 ng/ml) up to a time period of 72 h of exposure. However, a concentration- and time-dependent decrease in percent cell viability was observed after 12 h exposure of cells to LPS at higher concentrations (30–50 ng/ml). The most significant reduction in cell viability was observed in cells exposed to LPS at 50 ng/ml concentration for a period of 72 h, which was found to be approximately 50% of controls. Together, these results suggest that LPS exposure of ≤20 ng/ml fail to produce any significant toxicity to these adipocyte cells derived from human omentum tissues, and can be used without the concerns of LPS-induced effects.

### Transcriptional changes in human omentum tissues and adipocyte cells derived from human omentum tissues

To test our hypothesis that human omentum is capable of producing intrinsic anti-inflammatory response, we determined whether the major cytokines are induced in these tissues. Since our cytotoxicity data suggest that lower doses (5 and 10 ng/ml) of LPS do not significantly alter cell viability, we also treated adipocyte cells derived from human omentum tissues with these doses of LPS to determine whether cells are capable of inducing mRNA levels of specific cytokines even at these low doses. Our results are shown in Figure 2. We first compared the mRNA expression levels of inflammatory cytokines (IL-1β, IL-2, IL-4, IL-8, IL-10, TNF-α, and GM-CSF) of human omentum tissues with those of oral sub-mucosal tissues (Figure 2 a). Real Time PCR (qRT-PCR) was used to determine mRNA levels using specific primers (Table 1S) for each cytokine. It is evident from Figure 2 a that except for IL-2, IL-4 and IL-10, the levels of mRNA expression were significantly higher (∼2 fold or more; p≤0.01) for all other cytokines tested in omentum tissues when compared to oral sub-mucosal tissues. These results suggest that omentum tissues possess higher intrinsic level of inflammatory cytokines, which may be responsible for its wound healing properties. We further determined the levels of these cytokines in adipocyte cells derived from human omentum tissues and exposed to low non-toxic doses of LPS. Our results show that even at such low doses, LPS-induced alterations in the mRNA expression of genes associated with inflammatory cytokines were significantly higher. A dose- (5 and 10 ng/ml) dependent expression of these inflammatory cytokines in cells exposed to LPS for 48 h is shown in Figure 2 b. The expression levels were significantly higher for all the cytokines studied at 48 h in comparison to 24 h (data not shown for 24 h).

### Expression of anti-microbial/anti-bacterial peptide genes in human omentum tissues and adipocyte cells derived from human omentum tissues

To determine whether the levels of specific anti-microbial/anti-bacterial peptides are induced in human omentum, we carried out Real Time PCR (qRT-PCR) assay, using specific primers (Table S2) for LL-37, HNP 1-3, HBD-1, and HBD-2. The expression of genes associated with these antimicrobial activities were found to be significantly higher (2–3 fold; p≤0.01) in human omentum tissues compared to oral sub-mucosal tissues (Figure 3 a). Similar results were also obtained in adipocyte cells derived from human omentum tissues when exposed to low doses (5 and 10 ng/ml) of LPS for 48 h (Figure 3 b). These results clearly demonstrate that the expressions of anti-microbial/anti-bacterial peptides were significantly elevated in human omentum, which became more pronounced in the adipocyte cells derived from human omentum tissues.

### Translational changes in human omentum tissues and adipocyte cells derived from human omentum tissues

We further determined whether these alterations in the level of mRNA expressions of these cytokines are taking place at the level of protein expression. To determine the level of protein expressions of each of these cytokines (IL-1β, IL-2, IL-4, IL-8, IL-10, TNF-α, and GM-CSF), we carried out immunoreactions using specific antibodies. Our results (Figure 4 a & 4 b) showed that except for IL-4 and IL-10, protein expression of all other cytokines tested in omentum tissues when compared to oral sub-mucosal tissues were significantly higher (∼2 fold or more; p≤0.01). These results are having similar trend to those observed for mRNA levels (Figure 2 a); however, the magnitude of induction in the expression is higher. Results of western blot analyses in omentum-derived cultured cells exposed to LPS (5 and 10 ng/ml for 48 h) showed significant alteration in protein expressions for all cytokines tested including IL-2, IL-4 and IL-10 when compared to untreated cells (Figure 5 a–g). However, the increased levels of expressions of these cytokines were significantly higher than those of elevated levels observed in omentum tissues. Together, our results demonstrate that higher intrinsic levels of cytokines observed at the mRNA levels were translated into protein expression.

### Translational changes in the expression of antimicrobial/ anti-bacterial peptide gene proteins in human omentum tissues and adipocyte cells derived from human omentum tissues

Experiments were carried out to ascertain whether the alterations observed at transcriptional level for the expressions of antimicrobial/ anti-bacterial peptide gene are translational to the level of protein expression or not. To determine the level of protein expressions of each of these antimicrobial/ anti-bacterial peptide gene (LL-37, HNP1-3, hBD1 & hBD2), we carried out western blot analyses using specific antibodies. Our results (Figure 6 a) show significantly higher (∼2 fold or more; p≤0.01) protein expression of all antimicrobial/ anti-bacterial peptide tested in omentum tissues when compared to oral sub-mucosal tissues. These results show similar trends as observed for mRNA levels (Figure 3 a). Results of western blot analyses in omentum-derived cultured cells exposed to LPS (5 and 10 ng/ml for 48 h) showed significant alteration in protein expressions for all antimicrobial/ anti-bacterial peptide genes tested when compared to untreated cells (Figure 6 b–e). The levels of protein expressions of these antimicrobial/ anti-bacterial peptides in omentum derived cultured adipocytes were at par to the levels in observed in omentum tissues. Together, our results demonstrate that higher intrinsic levels of antimicrobial/ anti-bacterial peptides observed at the mRNA levels were translated into protein expression.

### Detection of secreted levels of cytokine profiles in adipocyte cells derived from human omentum tissues

We further analyzed the secreted cytokine profiles directly in cells. The results of cytokine expressions in the supernatants of cultures growing with or without LPS exposure are shown in Figure 7. Most significant induction in the level of TNF-α (49±1.3, 65±3.7 pg/ml) was observed following LPS (5 and 10 ng/ml) exposure for 24 h. Other cytokines showing significant increases were IL-1β (35±2.1, 50±3.2 pg/ml), IL-4 (31±2.6, 45±3.4 pg/ml), IL-8 (37±1.6, 55±2.8 pg/ml), and GM-CSF (22±1.4, 29±2.6 pg/ml), respectively at 24 h (Figure 7 a). The increased levels were sustainable for up to 48 h of exposure and levels of IL-2 (26±3.2, 37±3.4 pg/ml) and IL-10 (32±1.5, 36±2.3 pg/ml.) further increased at 48 h (Figure 7 b).

## Discussion

The wound healing properties of omentum have been well described [1], [2], [4]. The omentum also has a distinct role in the sealing of intestinal perforation in premature neonates [2] and in healing resistant sternal wounds (following infected sternotomy incisions) [6], [14]. Experimental studies have demonstrated that omentum can potentially prevent or reduce occurrence of infection following artificial aortic graft implantation [15], [16]. However, the underlying cellular and molecular mechanisms involved in the omentum- mediated prevention of infections and healing of injured organs remains poorly understood. We hypothesize that the high levels of pro- and anti-inflammatory cytokines under normal physiological conditions in the omentum (compared to the control), and under pathological conditions (a significant induction of cytokines and antimicrobial/antibacterial peptides) are likely to play an important role in the omental defense mechanism.

Our results confirm this pattern of expression of several cytokines and antimicrobial/antibacterial peptide in naïve omental tissue to support this hypothesis. Furthermore, induction of cytokines and anti-microbial peptides in the omentum appears exclusive to the adipocytes, since these effects were greatly enhanced in cultured adipocytes exposed to LPS. We identified the non cytotoxic doses of LPS (5 and 10 ng/ml) in isolated omentum cells. Our data of LPS cytotoxicity are in agreement with the study conducted by Melzig and Loose [17] on bovine aortic endothelial cells. Usually non- adipocyte cells or SV cells are considered to be the main source of pro-inflammatory adipose adipokine release by obese adipose tissue [7], [8], [9], [18], [19]. However, Bassols et al. [10] have shown that obese human omental differentiated adipocytes spontaneously release the pro-inflammatory cytokines IL-6 and MIF, and the chemokines IL-8, GRO, and MCP-1 [8], [10], [20]. Another study by Sopaskis et al. [21] showed that human subcutaneous adipose tissue even from non obese individuals release substantial amounts of IL-6, IL-8, and IL-1 RA and the gene expression of these cytokines, like that of IL-1β and PAI-1 is regulated by TNF-α. [21]. In the present investigations, the expression and up regulation of IL-1β, TNFα, IL-8, IL-2, IL-4 mRNAs and protein was recorded in non obese disease free omentum tissue as well as cultured adipocytes. We observed higher protein expression levels for tested cytokines compared to levels of mRNA expression in omentum tissues. It is well documented that cytokine mRNAs are expressed transiently and at low levels because they are tightly regulated and rapidly processed [22], [23], [24]. Whereas, the proteins of cytokines are known to express and accumulate in the cytoplasm and cell surface till secretion required [25]. Therefore, our results may not be an unusual phenomenon. The trends of Western blot analysis were in accordance with the result obtained by Fain et al. [7]. Our results show significant induction in the expression of IL-1β, TNF-α and IL-8 in cultured adipocytes following LPS (5 and 10 ng/ml) exposure for 24 and 48 h. Similar results have already been reported for the release of TNF α and IL-8 upon exposure of LPS in human differentiated omentum adipocytes [10]. It is well known that adipocytes express toll like receptor 4 (TLR-4) through which LPS activate intracellular inflammation pathways [26]. The role of cytokines-IL-8, TNFα and IL-1β has been suggested in the recruitment of monocytes into adipose tissue [21].

Antimicrobial peptides are effector molecules of innate immunity with microbicidal and pro- or anti-inflammatory activities. There is evidence that one such multifunctional peptide, LL-37, induces angiogenesis, a process essential for host defense, wound healing, and tissue repair [27], [28]. In normal tissue, these peptides have a negligible expression, but this may be triggered by injury or inflammation of the organ, and their expression or activation is essential for the organ to resist microbial infection [27]. Omental adipocytes could play a major role in protecting against infection by generating defensin (DEFA1-3) [5]. We also found that LPS exposure for 24 and 48 h induces significant expressions of LL-37 in omentum derived cultured adipocytes. To validate the expression of additional antimicrobial peptides in omental tissue, we evaluated the expression of HBD-1 and HBD-2, and found up regulation of these peptides at both m-RNA and protein levels. Our study has shown that normal (non diseased, non obese) human omentum has constitutive expression of several cytokines including (IL-1β, IL-4, IL-8, TNF-α, and GM-CSF), and antimicrobial peptides (LL-37, HNP-1, HBD-1 and 2). The cytokines and antimicrobial peptide surge is dose and time dependent (in response to LPS), and may be directly involved in the fight against bacteria.

In summary, for the first time, we demonstrate a significantly high expression (mRNA and protein) of selected pro- and anti-inflammatory cytokines, and antimicrobial peptides in normal human omentum tissue, when compared to control (human oral mucosal tissue). Previous reports have predominantly focused on cytokine expression in obese subjects [29]. Obese subjects have altered metabolism and are prone to a number of diseases, including cardiovascular disease and diabetes, and therefore, hardly are models to study wound healing. Moreover, previous studies did not evaluate a wide range of pro- and anti-inflammatory cytokines, and anti-microbial proteins, as was undertaken in the present study.

The expression levels of these cytokines and antimicrobial peptides were significantly higher in cultured omentum adipocytes than intact omentum tissue. LPS exposure (at non-toxic doses) to primary cultured adipocytes induced significantly higher levels of expression of cytokines, and anti-microbial peptides. This surge was seen to be LPS dose (5 and 10 ng/ml) and time dependent (24 and 48 h). Our data supports the view that these pro-inflammatory cytokines and antimicrobial peptides are involved in the wound healing properties of human omentum and that their expression is modulated by adipocytes. Together, our studies may provide a potential cellular and molecular mechanism for the defense mechanisms of omentum tissues in wound healing and infection, which in turn may have significant clinical applications.

## Materials and Methods

### Reagents and consumables

All the specified diagnostic kits were purchased from e-Biosciences Chemical Company Pvt. Ltd. St. USA. Culture medium DMEM F-12, antibiotics-antimycotic solution and fetal bovine serum were purchased from Gibco BRL, USA. Culture wares and other plastic consumables used in the study were procured commercially from Nunc, Denmark. Milli Q water (double distilled deionized water) was used in all the experiments. All the DNA primers and Lipopolysaccharide (LPS) were purchased from Sigma Aldrich, St. Louis, MO, USA.

### Ethical clearance for collection and transportation of human tissues

The protocol for human tissue collection was approved by the ‘Human Ethics Committee of Chhatrapati Shahuji Maharaj Medical University, Lucknow, India’. Six human omentum tissues were obtained during diagnostic laparoscopies having a negative outcome (after obtaining informed written consent from all the patients). All the patients were male between the age of 48.4±5.6 years (mean ± SE). They were non obese, free from malignancy or any other systemic disorder, and were not on any medication/medical treatments at the time of sample collection. Healthy oral mucosa (obtained from routine oral biopsies) was used as control. One part of tissue specimen collected was persevered and immediately transported to In Vitro Toxicology Laboratory, Indian Institute of Toxicology Research, Lucknow, India, for further processing. Tissues were collected in sterile Dulbecco's Modified Eagle's Medium (DMEM) supplemented with 10% fetal bovine serum (FBS) and antibiotic-antimycotic solution (Gibco BRL, USA), and immediately processed for isolation and cultivation of cells.

### Cell culture and exposure

Human omentum tissues (10 g) were cut in small pieces and placed in a sterile petridish containing a thin layer of minimal essential medium with 10% fetal bovine serum. The omentum tissues were fractioned into adipocytes and stromal vascular (SV) cells using 0.2% collagenase and 0.125% trypsin for 30 min at 37°C as described by Maury et al., 2007 [30]. Adipocytes were collected by centrifugation at 100×g for 5 min., the pellet of packed cells re-suspended in poly-L-lysine pre-coated six-well culture plates in complete minimal essential medium, and incubated at 37°C in an atmosphere of 95% air-5% CO_2_ for attachment. Growth was permitted to continue until cells attained a confluent monolayer, at which time they were trypsinzed (trypsin 0.05%–EDTA 0.53 mM) and passaged into T-25 culture flasks to expand cell population (First cell passage). Cells of third and fourth passages were trypsinzed and pooled for further experiments. The purity of adipocytes was checked at m-RNA levels using markers of macrophage (CD 68), endothelium (CD31) and adipocytes (leptin). Adipocytes fractions showing more than 95% purity were used for experiments. Cell numbers were determined using an Electronic Coulter Counter (Model Zf, Coulter Electronics, and Hialeah, FL, USA). Each batch of cells were assessed for viability using trypan blue dye exclusion test prior to experiments and only batches showing viability of more than 95% were used in the experiments.

### Experimental design

**Figure 1:**
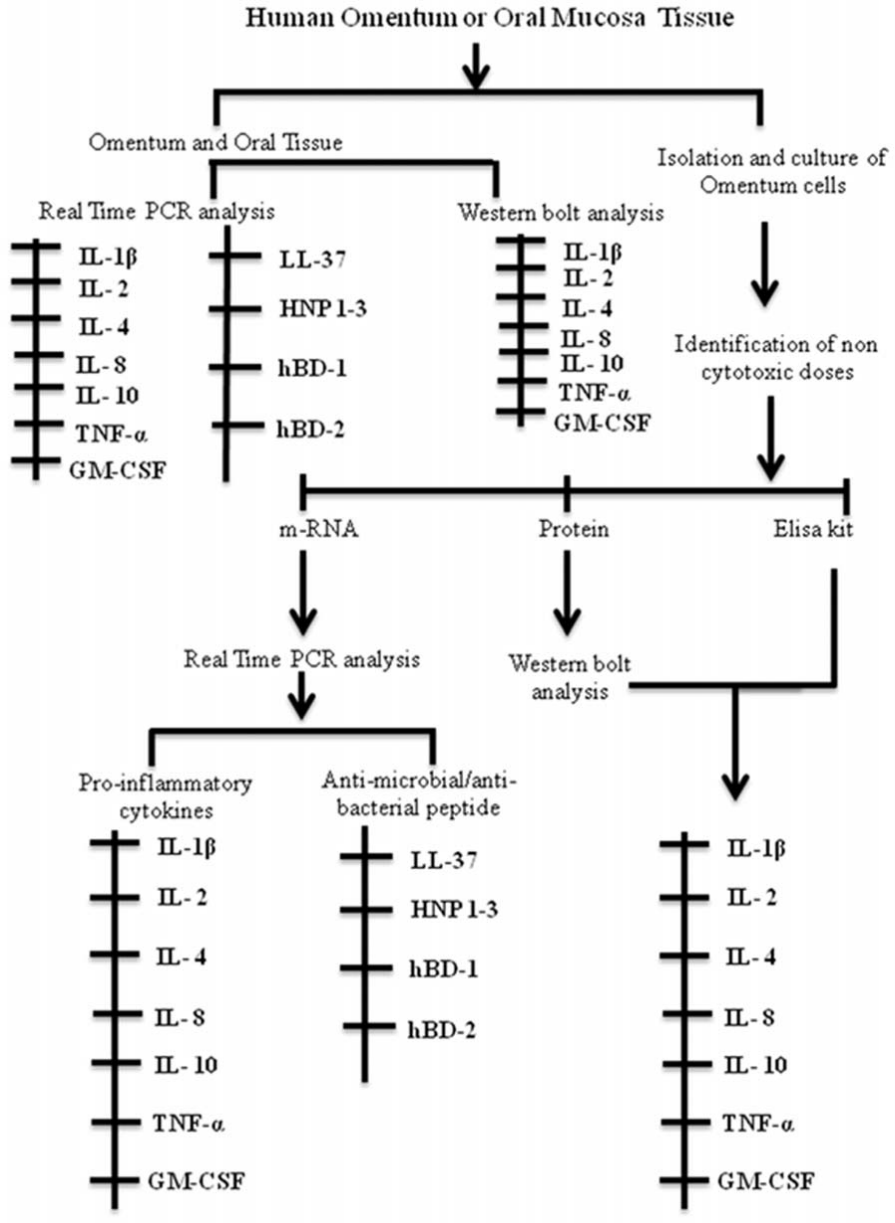
Schematic diagram showing experimental design.

Cultured adipocytes derived from human omentum were exposed to various concentrations (1–50 ng/mL) of LPS for 12–72 h to identify the non-cytotoxic doses. Levels of expression (mRNA and protein) were carried out for genes associated with pro- and anti-inflammatory cytokine responses and antibacterial/antimicrobial activity by exposing cultured cells to selected non-cytotoxic doses (5 and 10 ng/mL for 24 and 48) of LPS. Basal expressions of similar set of genes were also studied in whole omentum and human oral mucosal tissues (control) before and after exposing them to LPS. Entire analyses were done in triplicate for each of the six omental samples and similar set of control tissues. A schematic diagram of our experimental design is shown in figure 1.

**Figure 2:**
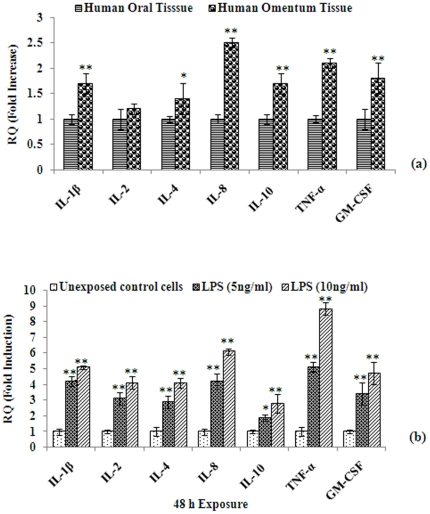
mRNA expression of inflammatory markers in human omentum tissue and cultured omental cells. Altered expression of mRNA of genes involved in inflammation in omentum tissue and compared with human oral tissue (Figure 2 a). Alterations expression of marker genes in omentum derived cultured adipocytes exposed to LPS for 48 h (Figure 2 b). Real Time quantitative PCR (RT^q^-PCR) was performed in triplicate by 2× Power SYBR Green PCR master mix. β-actin was used as internal control to normalize the data and LPS induced alterations in mRNA expression are expressed in fold change. Normal oral submucosal tissue was used to compare the changes in cytokines. *P<0.05- significant, **P<0.01- highly significant.

**Figure 3:**
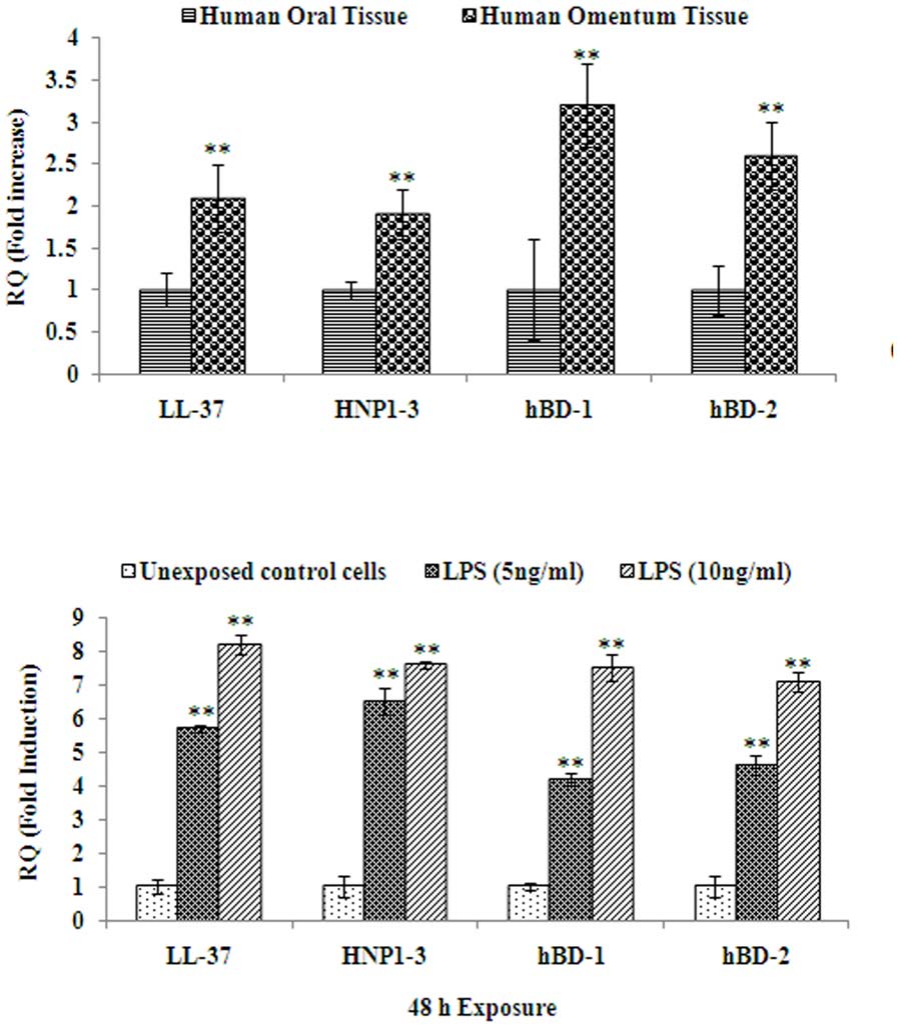
mRNA expression of antimicrobial/antibacterial peptide markers in human omentum tissue and cultured omental cells. Alerted expression of mRNA of antimicrobial peptide genes in omentum tissue and compared with human oral tissue (Figure 3 a). Alterations expression of marker genes in omentum derived cultured adipocytes exposed to LPS for 48 h (Figure 3 b). Real Time quantitative PCR (RT^q^-PCR) was performed in triplicate by 2× Power SYBR Green PCR master mix. β-Actin (ACTB) was used as internal control to normalize the data and LPS induced alterations in mRNA expression are expressed in fold change. Normal oral submucosal tissue was used to compare the changes in antimicrobial peptide activity. *P<0.05- significant, **P<0.01- highly significant.

**Figure 4:**
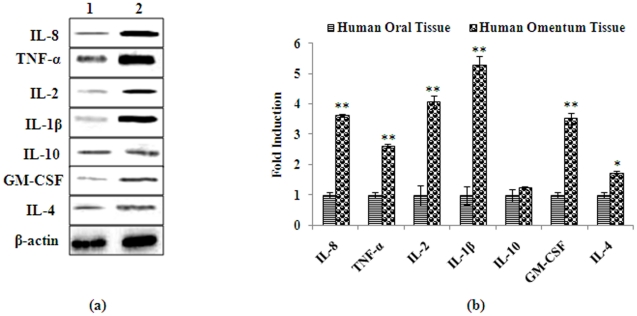
Protein expression of markers associated with inflammation in human omentum tissue. (a) Alterations in the expression of proteins involved in the induction of inflammatory cytokines in human omentum tissue. Normal oral submucosal tissue was used to compare the changes in protein of pro and anti-inflammatory cytokines (Figure 4 a). Lane (1): Normal oral submucosal tissue; Lane (2): Human omentum tissue. Molecular weight of protein studied: IL-1β (17 kDa), IL-2 (15 kDa) IL-4 (17 kDa), IL-8 (11 kDa), IL-10 (20 kDa), TNF-α (26 kDa) GM-CSF (16 kDa) and β-Actin (42 kDa) for normalization. (b) Relative quantification of alterations in the protein expression of cytokines in human omentum tissue. Normal oral submucosal tissue was used to compare the changes in protein of pro-inflammatory cytokines. β-Actin was used as internal control to normalize the data. Quantification (densitometry) was done in Gel Documentation System (Alpha Innotech, USA) with the help of AlphaEase™ FC StandAlone V.4.0 software. *P<0.05- significant, **P<0.01- highly significant.

**Figure 5:**
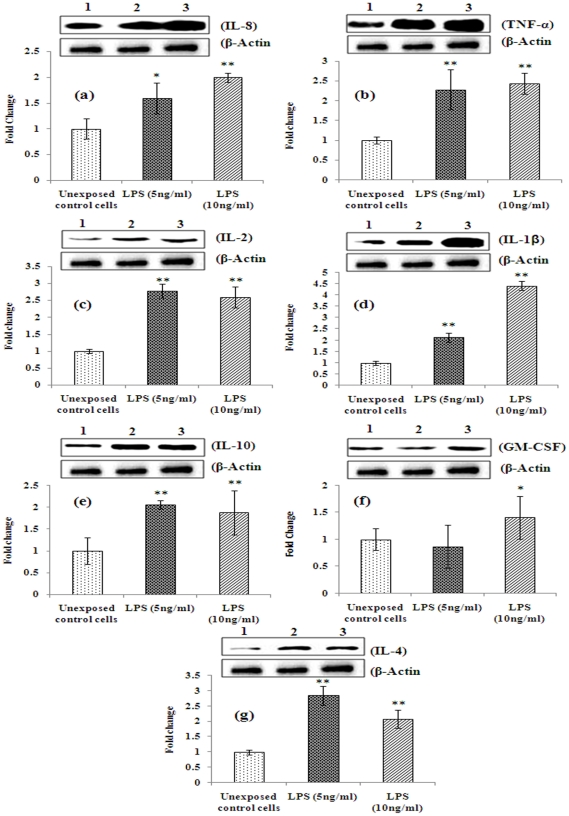
Constitutive expression and inducibility of proteins of inflammatory cytokines in primary cultures of omentum derived adipocytes exposed to LPS. Lane (1): Unexposed control cells; Lane (2): LPS (5 ng/ml) exposed omentum cells; (3) LPS (10 ng/ml) exposed omentum cells. Molecular weight of protein studied: IL-1β (17 kDa), IL-2 (15 kDa) IL-4 (17 kDa), IL-8 (11 kDa), IL-10 (20 kDa), TNF-α (26 kDa) GM-CSF (16 kDa) and β-actin (42 kDa) for normalization. β-Actin was used as internal control to normalize the data. Quantification (densitometry) was done in Gel Documentation System (Alpha Innotech, USA) with the help of AlphaEase™ FC StandAlone V.4.0 software. *P<0.05- significant, **P<0.01- highly significant.

**Figure 6:**
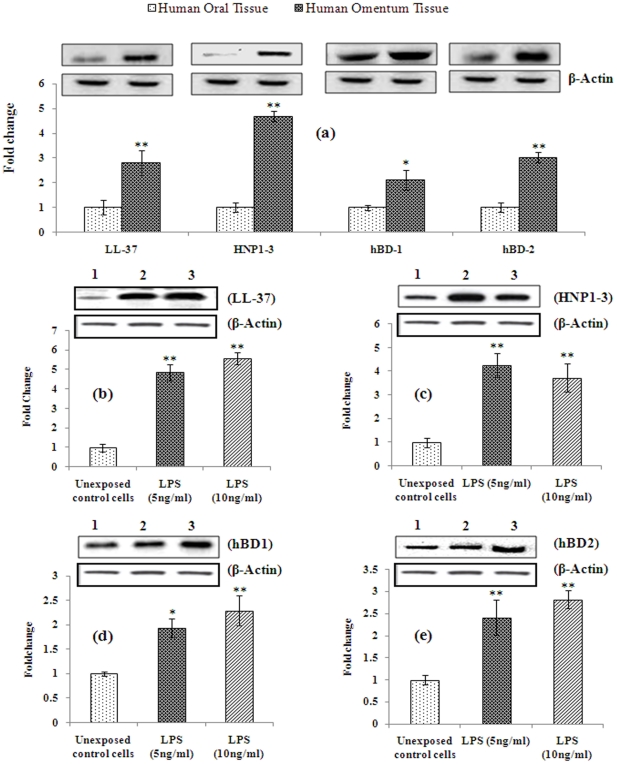
Alterations in the protein expression of antimicrobial/antibacterial peptides in omentum tissue and omentum derived adipocytes. Normal oral submucosal tissue was used to compare the protein expression (Figure 6 a). Constitutive and inducibility in the protein expression of antimicrobial/antibacterial peptide genes in omentum derived adipocytes exposed to LPS (Figure 6 b–e). The expression levels of antimicrobial/antibacterial peptides tested are: LL-37 (20 kDa), HNP1-3 (∼4 kDa) hBD-1 (∼4 kDa), hBD-2 ( ∼5 kDa) and β-Actin (42 kDa). β-Actin was used as internal control to normalize the data. Quantification (densitometry) was done in Gel Documentation System (Alpha Innotech, USA) with the help of AlphaEase™ FC StandAlone V.4.0 software. *P<0.05- significant, **P<0.01- highly significant.

**Figure 7:**
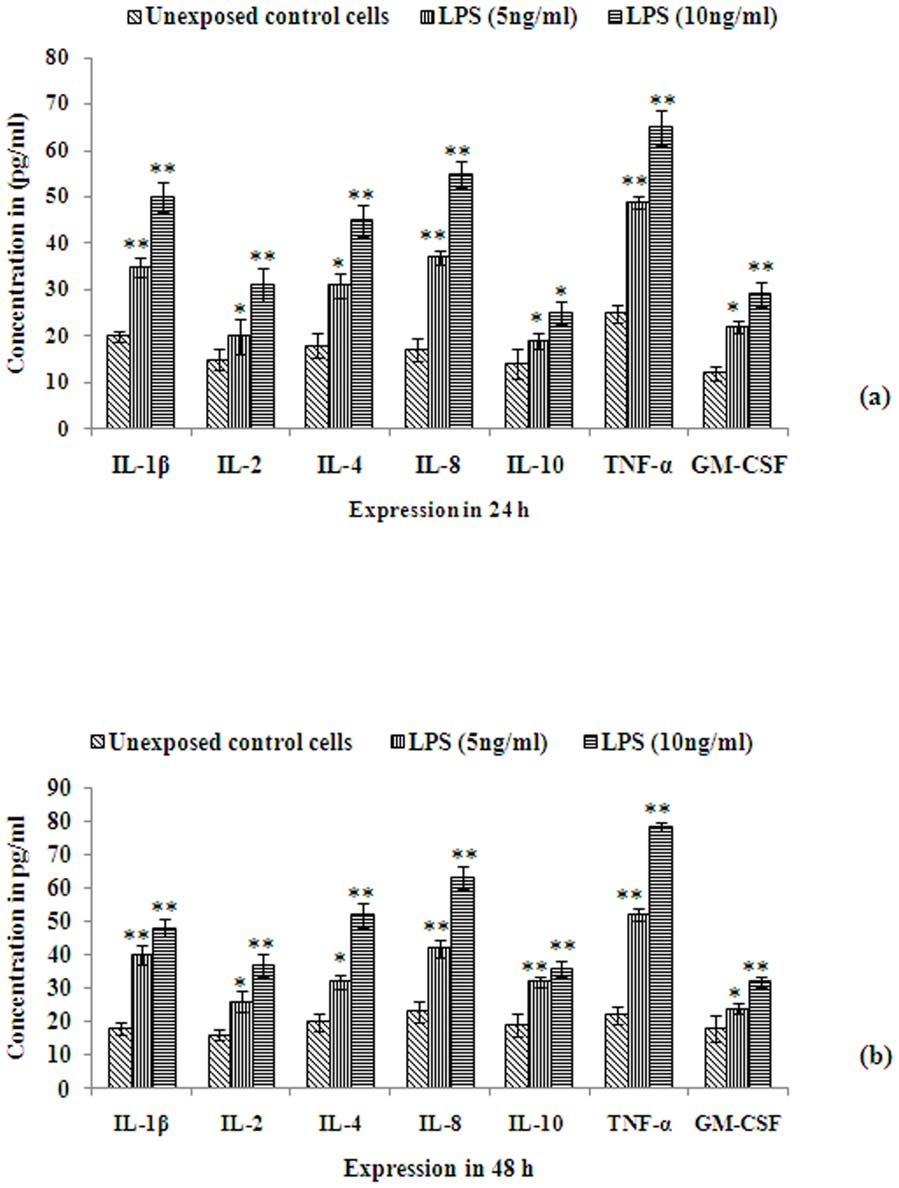
Functional activity assay for inflammatory cytokines in human omentum derived cultured adipocytes. Alterations in the activity of inflammatory cytokines in isolated cultured omentum cells and LPS induced omentum cells for 24 h (Figure 7 a) and 48 h (Figure 7 b) through ELISA set go kit (e-Biosciences, USA). Data were analyzed in pg/ml through preparation of stander curve as supplied by manufacture.

### Cytotoxicity assessment (MTT assay)

Non-cytotoxic doses of LPS were assessed using Tetrazolium bromide salt (MTT) assay. The assay was carried out using the protocol described earlier by Srivastava et al. [31]. In brief, cells (1×10^4^) were allowed to adhere for 24 h under high humid environment in 5% CO_2_- 95% atmospheric air at 37°C in 96 well culture plates. The medium was aspirated and cells were exposed to fresh medium containing LPS (1–50 ng/ml) for 12–72 h. Tetrazolium bromide salt (5 mg/ml of stock in PBS) was added (10 µl/well in 100 µl of cell suspension) and plates were further incubated for 4 h. At the end of the incubation period, the reaction mixtures were carefully taken out and 200 µl of DMSO added to each well. The plates were kept on rocker shaker for 10 min at room temperature and then analyzed at a wavelength of 550 nm using Multiwell Microplate Reader (Synergy HT, Bio-Tek, USA). Parallel sets of unexposed cells were also run under identical conditions and used as basal controls.

### Real Time-PCR studies

The study of pro-inflammatory and antimicrobial/antibacterial peptide genes expression at transcriptional level was done by SYBR Green quantitative Real Time PCR as earlier describe by us [32]. Total RNA was isolated from cultured cells, omentum tissues, and oral mucosal tissues using the TRIzol method (Invitrogen). The total amount of RNA was determined using Nanodrop (ND1000) Spectrophotometer and purity was assessed by denaturing agarose gel electrophoresis. cDNA synthesis was carried out with 1 µg of RNA using High Capacity cDNA Reverse Transcription Kit (Applied Biosystems, USA). Quantitative Real Time PCR was performed using ABI prism 7900HT system (Applied Biosystems, USA). Real time assay reactions were carried out with 2× Power SYBR Green PCR master mixes (Applied Biosystems, USA) as per the protocol provided by manufacturer. For PCR amplification, an initial step of 50°C for 2 min was performed followed by denaturation step of 95°C for 15 min. Then 45 cycles of denaturation (95°C for 15 Sec) and annealing and extension step (60°C for 60 Sec) were performed. PCR reactions were carried out in triplicates for each sample. Dissociation reaction was also carried out for each primer to check the specificity of primers. The comparative Ct method for relative quantification (ΔΔCt method), which describes the change in expression of the target gene in a test sample relative to a calibrator sample, was used to analyze the data. Data were analyzed using 7900HT Sequence Detector System (SDS) software version 2.2.1 (Applied Biosystems, USA). Results were expressed relative to the housekeeping gene (β-actin). Details of primers used for specific genes are given in Table S1 and Table S2).

### Western Blot Analyses

Levels of protein expression were carried out using Western immunoblotting as describe earlier by us [33]. The LPS-induced translational changes in the level of expression of selected pro-inflammatory cytokines (IL-1β, IL-2, IL-4, IL-8, IL-10, TNF-α, GM-CSF) and antimicrobial/antibacterial peptide (LL-37, HNP 1-3, Hbd-1 and Hbd-2) were determined following exposure of LPS (5 and 10 ng/ml) for 48 h. The same analyses were also carried out in the tissue samples (with and without LPS exposure) to measure constitutive expression of cytokines. Cells were pelleted and lysed using CelLytic™ M Cell Lysis Reagent (Sigma, USA) in the presence of protein inhibitor cocktail (Sigma, USA). Protein concentrations were estimated using BCA Protein Assay Kit (Lamda Biotech, Inc., St. Louise, MO, USA). Samples containing 50 µg/well of protein were run on 10–14% Tricine-SDS gel. After electrophoresis, gels were transferred onto Immobilon-P membrane (Millipore, USA). Nonspecific binding was blocked with 5% nonfat dry milk powder in TBST buffer for 2 h at 37°C. After blocking, the membranes were incubated overnight at 4°C with anti-protein primary antibodies specific for IL-1β, IL-2, IL-4, IL-8, IL-10, TNF-α, GM-CSF, LL-37, HNP 1-3, Hbd-1, or Hbd-2 (R&D Systems, USA) in blocking buffer. The membrane was then incubated for 2 h at room temperature with secondary antibody conjugated with horseradish peroxidase (Calbiochem, USA). The blots were developed using TMB-H_2_O_2_ (Sigma, USA) and densitometry for protein specific bands were carried out using the Gel Documentation System (Alpha Innotech, USA) with the help of AlphaEase™ FC StandAlone V.4.0 software.

### Cytokine determination in culture cells

Commercially available “Ready-SET-Go! ELISA Kit” (e Biosciences, USA) was used to determine the levels of 7 cytokines in the LPS-induced cultured omentum cells. These cytokines include IL-1β, IL-2, IL-4, IL-8, IL-10, TNF-α, and GM-CSF. In brief, cells were exposed to 5 and 10 ng/ml LPS for 24 and 48 h. At the end of exposure period, the cell supernatant (100 µl) in triplicate wells were used for the determination of various cytokines as per manufacturer's instructions. The plates were then analyzed at 450 nm using Multiwell microplate reader (Synergy HT, Bio-Tek, USA). Untreated sets were also run under identical conditions and served as basal control.

### Statistical analyses

Results are expressed as the mean (SEM) from the values obtained from at least three independent experiments, and triplicate samples were used in each experiment. Statistical analyses were performed using one-way analysis of variance (ANOVA) followed by post hoc Dunnett's test to compare the findings in different groups. The values, *p<0.05, were considered significant and **P<0.01 highly significant.

## Supporting Information

Figure S1 **Cytotoxicity assay.** Identification of non-cytotoxic doses of LPS in human omentum cells assessed by standard endpoints viz., MTT Assay. Data represent as mean ± S.E.M. of triplicate. *P<0.05- significant, **P<0.01- highly significant.
(TIF)

Table S1 **Real Time primer sequences of genes for tested cytokines.**
(TIF)

Table S2 **Real Time primer sequences of genes for tested antimicrobial/ antibacterial peptides.**
(TIF)
