# Supplementary figures and images for: The Anti-Inflammatory and Antibacterial Basis of Human Omental Defense: Selective Expression of Cytokines and Antimicrobial Peptides
Source: PLoS One. 2011 May 24;6(5):e20446. doi: 10.1371/journal.pone.0020446 (PMC3101256; doi:10.1371/journal.pone.0020446)

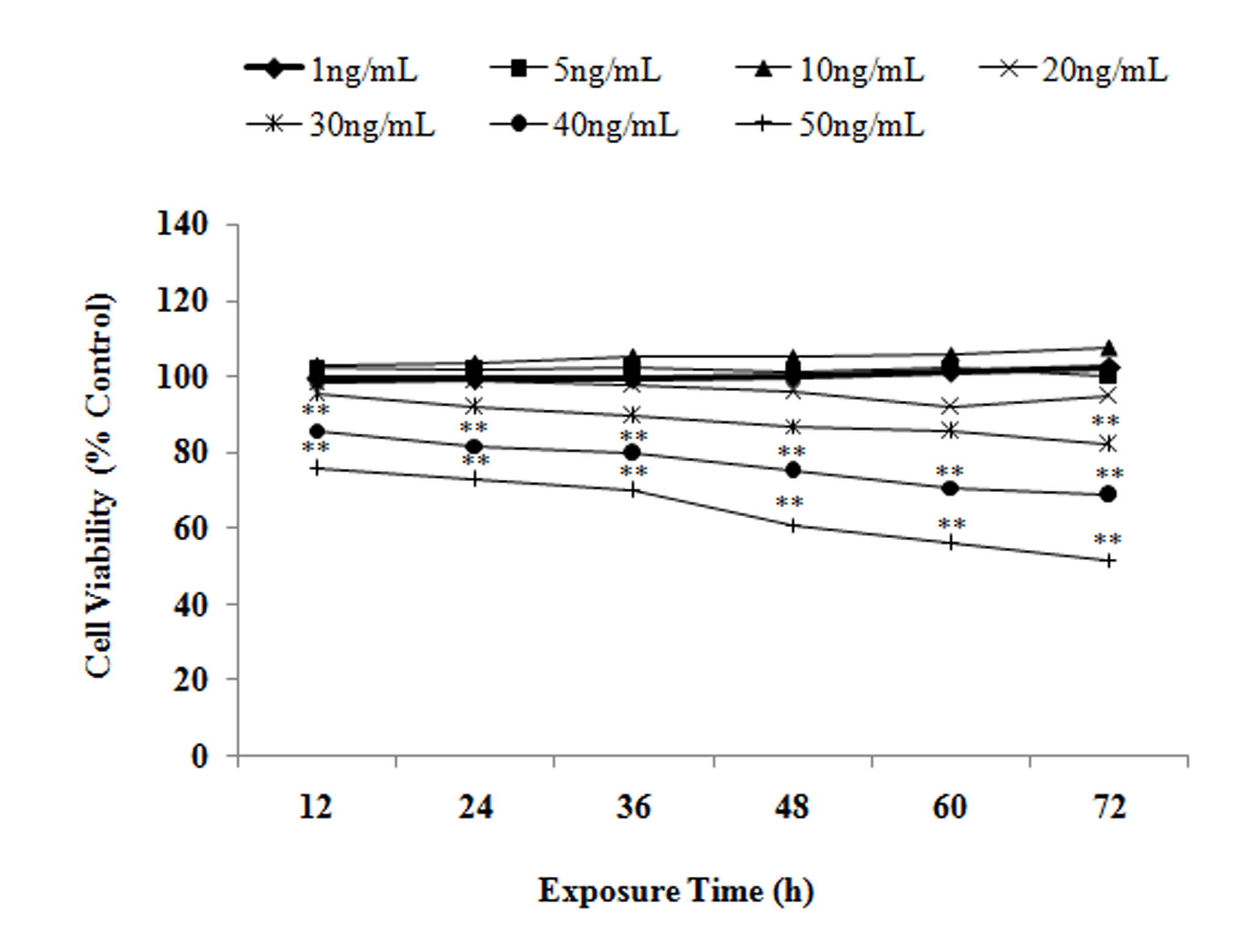

Supplement: Figure S1 — Cytotoxicity assay. Identification of non-cytotoxic doses of LPS in human omentum cells assessed by standard endpoints viz., MTT Assay. Data represent as mean ± S.E.M. of triplicate. *P<0.05- significant, **P<0.01- highly significant. (TIF) [file pone.0020446.s001.tif]

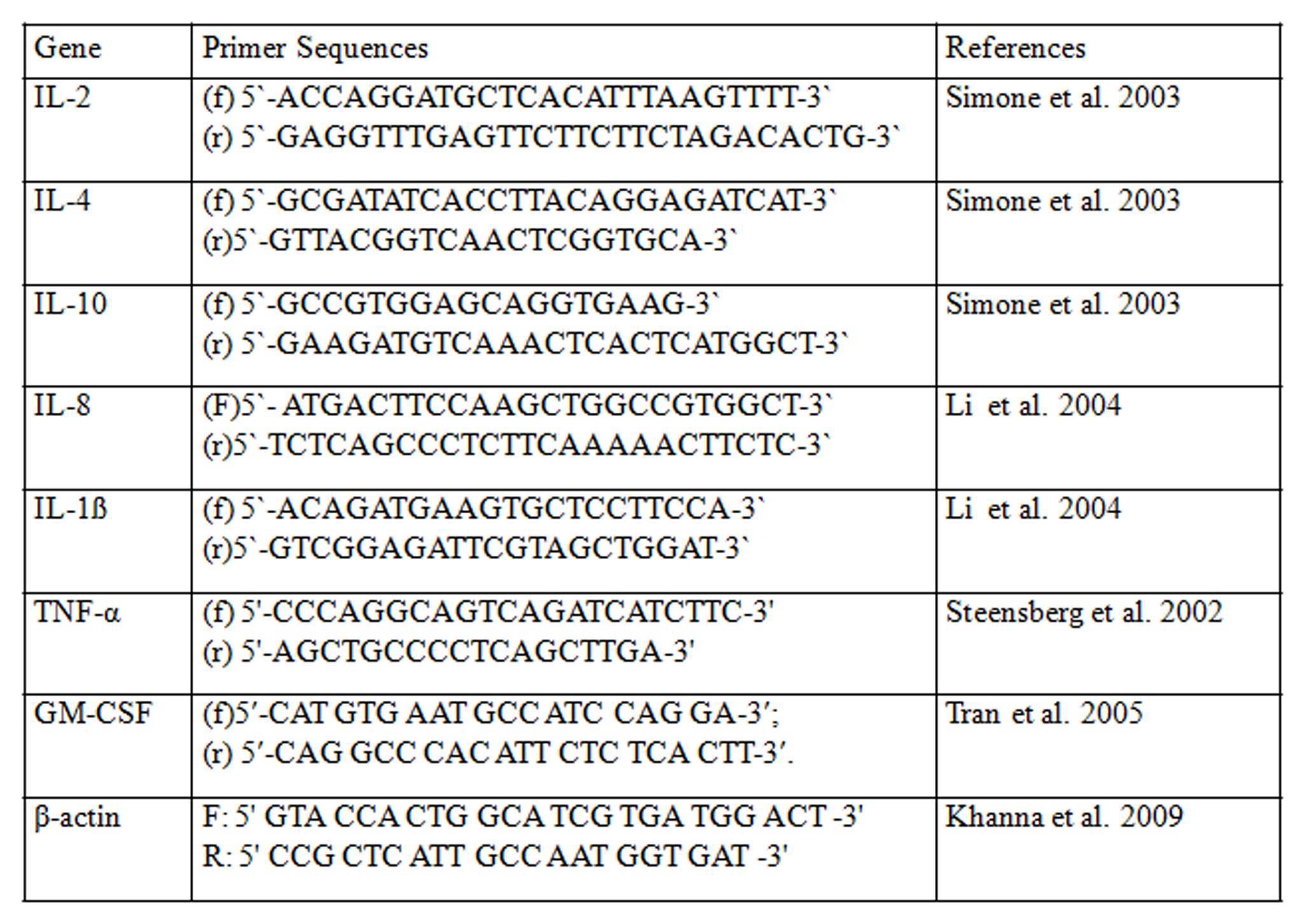

Supplement: Table S1 — Real Time primer sequences of genes for tested cytokines. (TIF) [file pone.0020446.s002.tif]

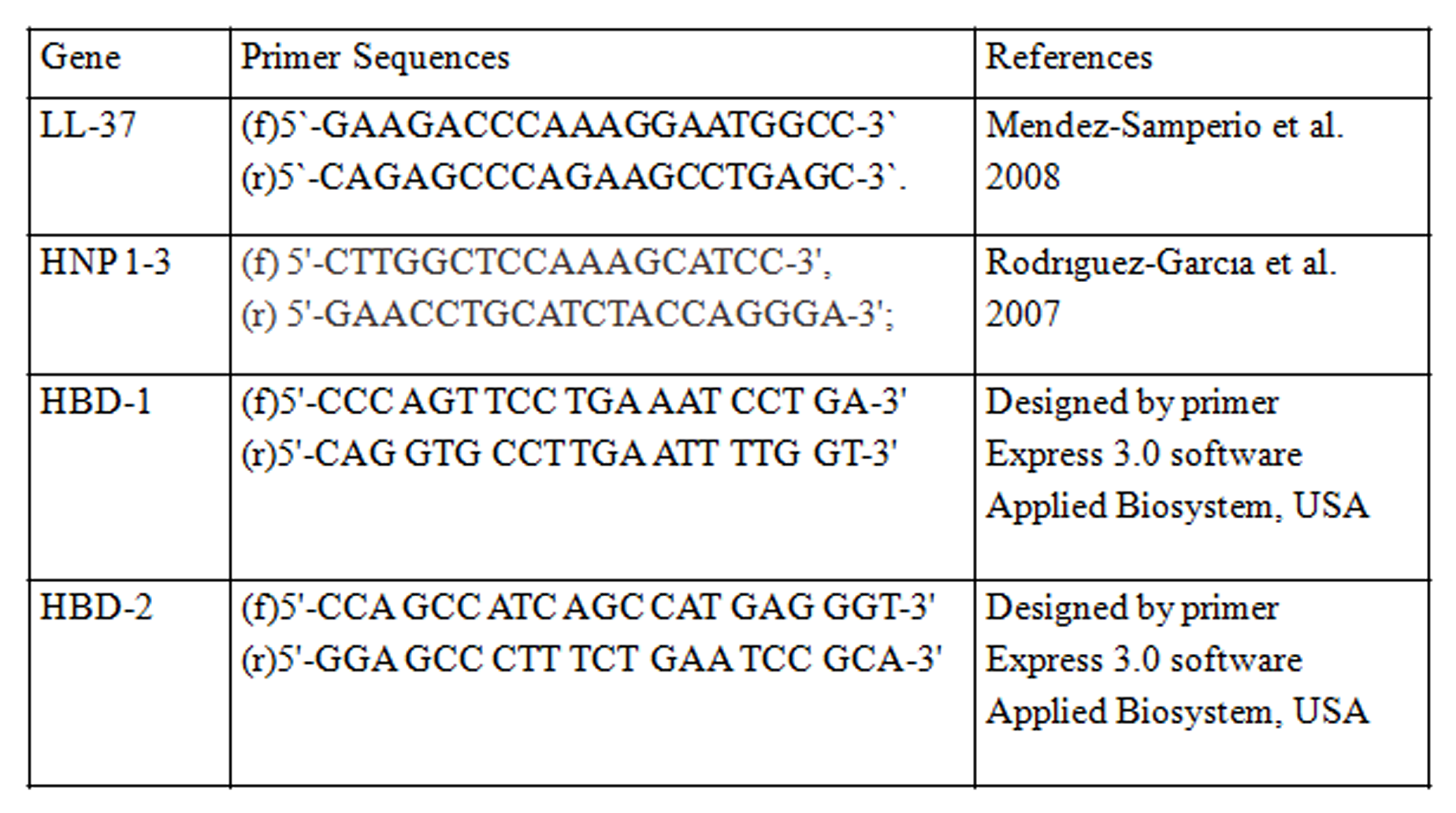

Supplement: Table S2 — Real Time primer sequences of genes for tested antimicrobial/ antibacterial peptides. (TIF) [file pone.0020446.s003.tif]
